# Interleukin 6 trigged ataxia-telangiectasia mutated activation facilitates lung cancer metastasis via MMP-3/MMP-13 up-regulation

**DOI:** 10.18632/oncotarget.5825

**Published:** 2015-10-22

**Authors:** Yi Na Jiang, Hong Qiong Yan, Xiao Bo Huang, Yi Nan Wang, Qing Li, Feng Guang Gao

**Affiliations:** ^1^ Department of Immunology, Basic Medicine Science, Medical College, Xiamen University, Xiamen 361005, People's Republic of China; ^2^ State Key Laboratory of Oncogenes and Related Genes, Shang Hai Jiao Tong University, Shanghai 200032, People's Republic of China

**Keywords:** ataxia-telangiectasia mutated, interleukin 6, lung cancer, metastasis, matrix metalloproteinases

## Abstract

Our previous studies show that the phosphorylation of ataxia-telangiectasia mutated (ATM) induced by interleukin 6 (IL-6) treatment contributes to multidrug resistance formation in lung cancer cells, but the exact role of ATM activation in IL-6 increased metastasis is still elusive. In the present study, matrix metalloproteinase-3 (MMP-3) and MMP-13 were firstly demonstrated to be involved in IL-6 correlated cell migration. Secondly, IL-6 treatment not only increased MMP-3/MMP-13 expression but also augmented its activities. Thirdly, the inhibition of ATM phosphorylation efficiently abolished IL-6 up-regulating MMP-3/MMP-13 expression and increasing abilities of cell migration. Most importantly, the *in vivo* test showed that the inhibition of ATM abrogate the effect of IL-6 on lung cancer metastasis via MMP-3/MMP-13 down-regulation. Taken together, these findings demonstrate that IL-6 inducing ATM phosphorylation increases the expression of MMP-3/MMP-13, augments the abilities of cell migration, and promotes lung cancer metastasis, indicating that ATM is a potential target molecule to overcome IL-6 correlated lung cancer metastasis.

## INTRODUCTION

Multidrug resistance (MDR) formation and metastasis are the important issues for lung cancer therapeutic failure [1]. Tumors [2–5] or stromal cells [6–8] expressing interleukin 6 (IL-6) have been documented to promote tumor metastasis [1, 9–10]. Our previous study reveals that IL-6 contribute to lung cancer chemotherapeutic resistance [11]. The good correlation of IL-6 level and poor clinical outcome of lung cancer, breast cancer and pancreatic cancer patients indicates that IL-6 might be a pivotal molecule to overcome inflammation-correlated lung cancer metastasis [12–13].

Matrix metalloproteinases (MMPs) mediated degradation of the extracellular matrix (ECM) is an initial step for metastasis. MMP-3 has been revealed to remodel ECM [14–15] and has a close correlation with the progression of breast, gastric and lung cancer [16–18]. Meanwhile, MMP-13 was also documented to be activated by MMP-3 and contribute to metastasis [19–21]. The activities of MMP-3/MMP-13 could be increased by TNF-α treatment and mediated TNF-α augmented metastasis [22]. But until now, little is known about the roles of MMP-3 and MMP-13 in IL-6 correlated lung cancer metastasis.

Ataxia-telangiectasia mutated (ATM) is a serine/threonine kinase that is activated by DNA double strand break [23]. The treatment with IL-6 also triggers ATM phosphorylation without apparent DNA damage [11]. The phosphorylation of ATM up-regulates MDR-associated protein expression, and contributes to chemotherapeutic resistance [24–25]. The effect of IL-6 on ATM raises the question of whether ATM activation is involved in IL-6 correlated lung cancer metastasis. Nevertheless, little is known about the role of ATM phosphorylation in IL-6 increased expressions of MMP-3 and MMP-13.

In the present study, we found that the high IL-6 level reveals both the increased expression of MMP-3/MMP-13 and the enhanced migration abilities of lung cancer cells. Then, the inhibition of ATM phosphorylation abolishes IL-6 increased expression of MMP-3/MMP-13, hence abrogates IL-6 correlated lung cancer metastasis both *in vitro* and *in vivo*. All these findings demonstrate that IL-6 inducing ATM activation increases the expression of MMP-3/MMP-13, augments the abilities of cell migration and promotes lung cancer metastasis, indicating that ATM is a potential target molecule to overcome IL-6 correlated lung cancer metastasis.

## RESULTS

### IL-6 level correlates cell migration abilities in lung cancer cells

**Figure 1:**
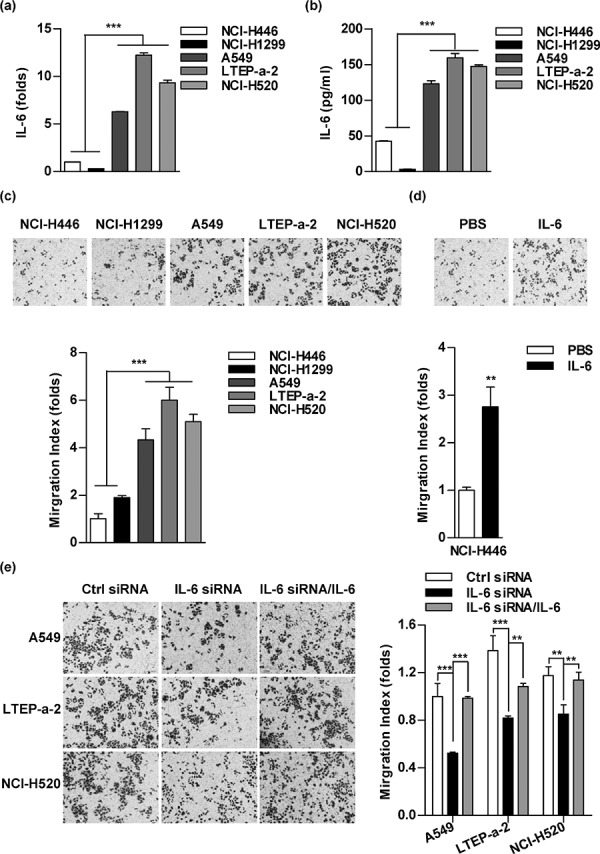
The level of IL-6 correlates to the abilities of cell migration in lung cancer cells The cellular RNA and supernatant of indicated lung cancer cells were prepared and the expression of IL-6 was determined by RT-qPCR **a.** and ELISA **b.** A panel of lung cancer cells was treated with PBS **c.** IL-6 (5 ng/ml) **d.** or IL-6 siRNA transfection/IL-6 **e.** and the cell migration was determined via Transwell migration assay by calculating the number of migrated cells in three visual fields. The data are presented as the mean ± SEM, *n* = 3. **p < 0.01, ***p < 0.001, Student's *t* test or one-way ANOVA with post Newman-Keuls test. One representative from three experiments is shown.

To explore the effect of IL-6 on cell migration, we firstly determined IL-6 levels in a panel of lung cancer cells. Contrast to NCI-H446 and NCI-H1299 cells, there is a relatively higher IL-6 level in A549, LTEP-a-2 and NCI-H520 cells (Figure 1a–1b). Consistent with the higher IL-6 level, the more migration cells were observed in A549, LTEP-a-2 and NCI-H520 cells (Figure 1c). When NCI-H446 cells were replenished with IL-6, the ability of cell migration increased accordingly (Figure 1d). Meanwhile, the down-regulation of IL-6 in A549, LTEP-a-2 and NCI-H520 cells led to a significant decline of migration cells (Figure 1e). As IL-8 was reported to affect cell migration by mediating angiogenic activity [26], we next detected the IL-8 level in lung cancer cells. No difference was found between NCI-H446 and A549 cells (Supplementary Figure S1a). As the increased migration cells is usually yielded by promoted cell proliferation or augmented cell migration, the observation that IL-6 had no effect on cell proliferation excludes the possibility that IL-6 increases cell migration by promoting proliferation (Supplementary Figure S1b–S1c). The above results demonstrate that the IL-6 level correlates cell migration abilities in lung cancer cells.

### MMP-3/MMP-13 is involved in IL-6 increasing cell migration in lung cancer cells

**Figure 2:**
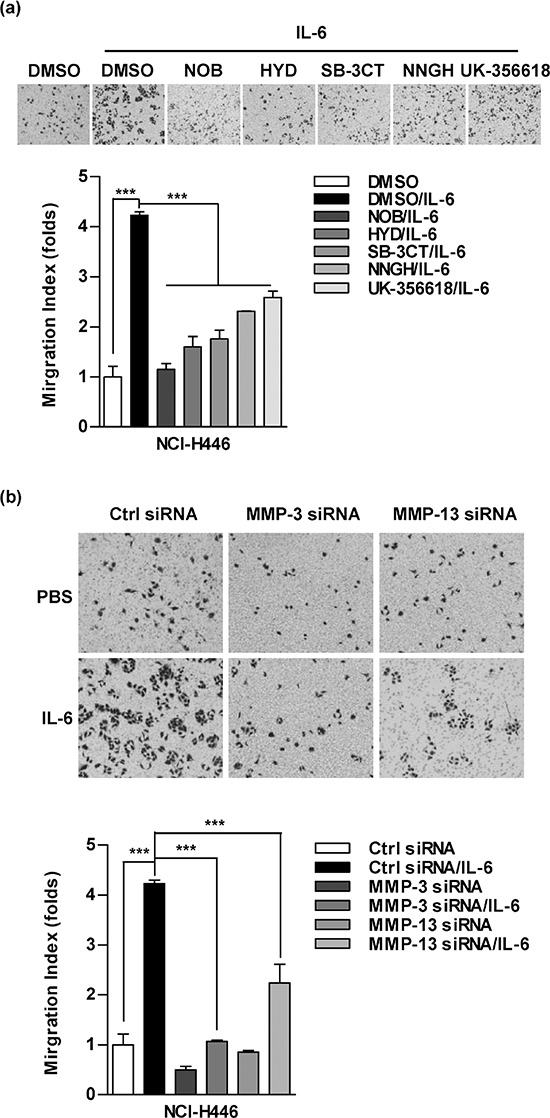
MMPs are involved in interleukin 6 (IL-6) increasing cell migration in lung cancer cells NCI-H446 **a.** or A549 **b.** cells were conferred the treatment with Nobiletin (NOB, 64 μM), N-CBZ-Pro-Leu-Gly hydroxamate (HYD, 50 μM), SB-3CT (10 μM), NNGH (10 μM), UK-356618 (73 nM) (a) or MMP-3/MMP-13 siRNA transfection (b), respectively, which were further stimulated with IL-6 (5 ng/ml). The ability of cell migration was determined by Transwell migration assay. The data are presented as the mean ± SEM, *n* = 3. ***p < 0.001, One-way ANOVA with post Newman-Keuls test. One representative from three experiments is shown.

MMPs activities and epithelial-mesenchymal transition (EMT) are the main landmarks of metastasis. To investigate the roles of MMPs in IL-6 increased cell migration, MMPs inhibitors were used and cell migration was determined. Both the broad-spectrum inhibitor (NOB) and the specific inhibitors, such as HYD (MMP-1), SB-3CT (MMP-2), NNGH (MMP-3) and UK-356618 (MMP-13), efficiently abolished the effect of IL-6 on cell migration in NCI-H446 (Figure 2a) and A549 cells (Supplementary Figure S2). As MMP-3/MMP-13 plays the pivotal roles in TNF-α increased metastasis [22], we therefore investigated the role of MMP-3/MMP-13 in IL-6 enhanced lung cancer metastasis. As shown in Figure 2b, the silencing of MMP-3 and MMP-13 abrogated the effect of IL-6 on cell migration in NCI-H446 cells. Together, these data demonstrate that MMP-3 and MMP-13 are the key MMPs in IL-6 promoting lung cancer metastasis.

### IL-6 increases MMP-3/MMP-13 expression and activity in lung cancer cells

**Figure 3:**
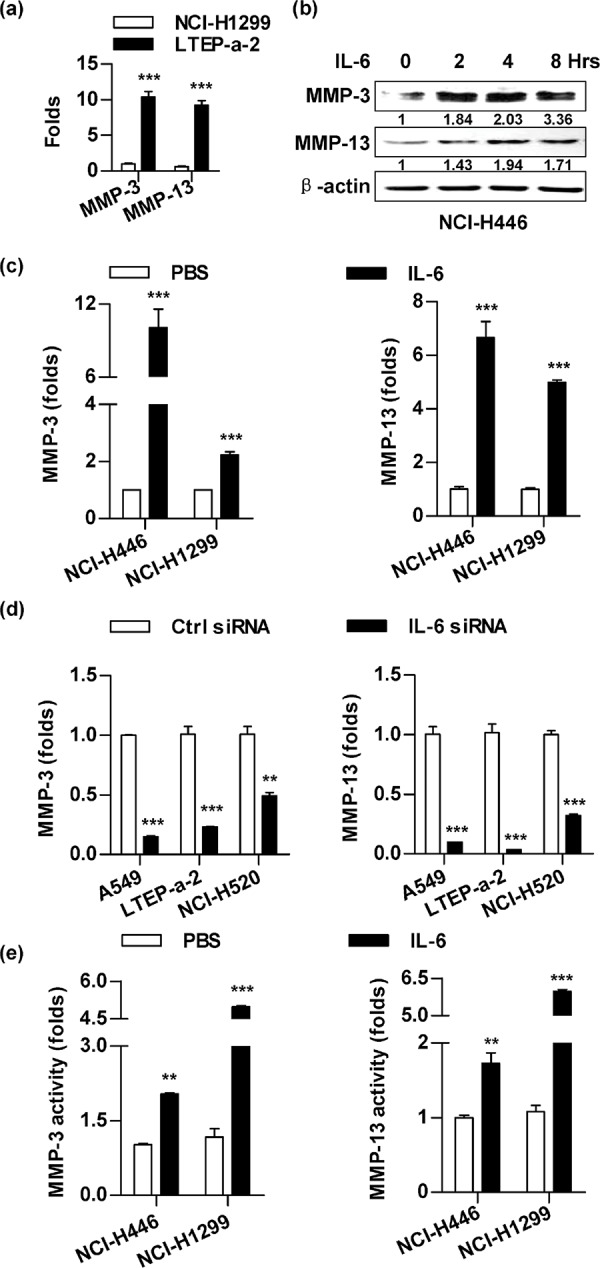
The treatment with IL-6 promotes the expressions and activities of MMP-3/MMP-13 in lung cancer cells A panel of lung cancer cells was conferred the treatment with PBS **a.** IL-6 (5 ng/ml) **b–c, e.** or IL-6 siRNA transfection d. The effects of IL-6 on expression (b-d) and activities (e) of MMP-3/MMP-13 were determined by RT-qPCR (a, c-d), western blot (b) and ELISA (e), respectively. The relative band intensity of MMP-3/MMP-13 in western blot was quantified using IMAGE J software and indicated under each lane. The data are presented as the mean ± SEM, *n* = 3. **p < 0.01, ***p < 0.001, Student *t* test. One representative from three experiments is shown.

As the cells that had higher level of IL-6 (Figure 1) also revealed higher MMP-3/MMP-13 expression (Figure 3a), we next explored the effect of IL-6 on MMPs' expression by IL-6 replenishment or siRNA silencing. The IL-6 replenishment increases MMP-3/MMP-13 expression not only in protein level (Figure 3b), but also in mRNA level (Figure 3c) in NCI-H446 and NCI-H1299 cells. On the other hand, MMP-3/MMP-13 expression can be decreased by IL-6 silencing in A549, LTEP-a-2 and NCI-H520 cells (Figure 3d). Importantly, the activities of MMP-3/MMP-13 were also increased by IL-6 replenishment (Figure 3e). Apart from MMP-3/MMP-13, the expression of MMP-1/MMP-2 was also found to correlate with the level of IL-6 (Supplementary Figure S3). All these results indicate that IL-6 increases MMPs expressions and activities in lung cancer cells.

### Inhibition of ATM phosphorylation abrogates the effect of IL-6 on cell migration in lung cancer cells

**Figure 4:**
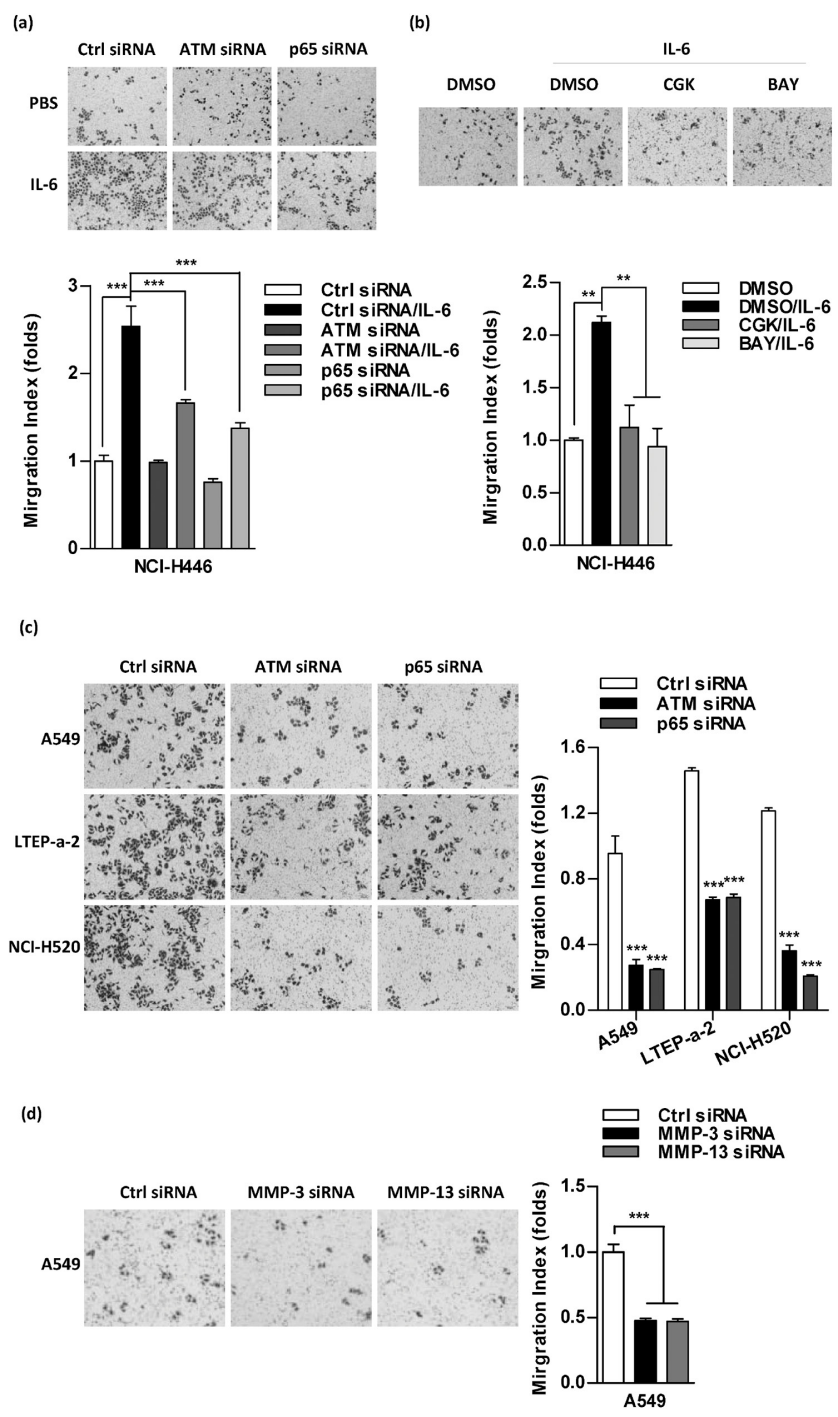
Inhibition of ATM and NF-κB activation abrogates the effect of IL-6 on cell migration in lung cancer cells To investigate the role of ATM, NF-κB and MMP-3/MMP-13 in IL-6 increasing cell migration, the indicated cells were performed siRNA transfection **a, c, d.** or kinases inhibitor treatment **b.** and cell migration was determined by Transwell migration assay. The data are presented as the mean ± SEM, *n* = 3. ***p < 0.001, One-way ANOVA with post Newman-Keuls test. One representative from three experiments is shown. CGK733 and BAY11–7082 were shown as CGK and BAY, respectively, for limited space in the figure.

Despite the effects of IL-6 in ATM phosphorylation [11] and in tumor invasion [9–10] were documented respectively, the exact role of ATM phosphorylation in IL-6 increasing lung cancer metastasis is still unknown. Indeed, both the silencing of ATM/p65 (Figure 4a) and the inhibition of relative kinases (Figure 4b, Supplementary Figure S4) abrogated IL-6's effect on cell migration in NCI-H446 cells. The silencing of ATM/p65 in A549, LTEP-a-2 and NCI-H520 cells also decreased the ability of cell migration (Figure 4c). As the silencing of these genes had no effect on cell proliferation (Supplementary Figure S5a–S5b) and down-regulation of MMP-3/MMP-13 inhibited cell migration (Figure 4d), the inhibition of ATM phosphorylation abolishing IL-6's effect on cell migration indicates that the phosphorylation of ATM is involved in IL-6 increasing lung cancer metastasis.

### Inhibition of ATM phosphorylation abrogates IL-6 increasing MMP-3/MMP-13 expression

**Figure 5:**
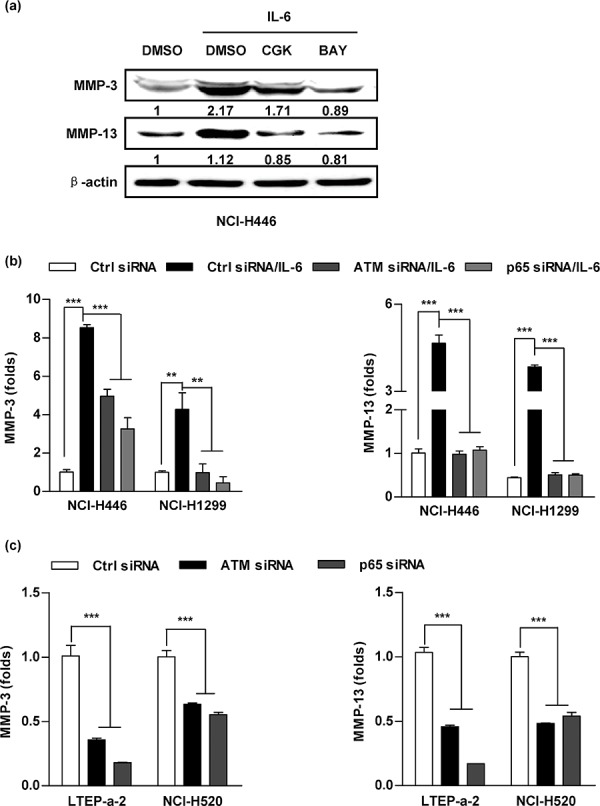
Inhibition of ATM and NF-κB activation abrogates the effect of IL-6 on MMP-3/MMP-13 up-regulation A panel of lung cancer cells was pretreated with inhibitors of ATM (CGK733, 20 μM), p65 (BAY11–7082, 20 μM) **a.** or siRNA transfection **b–c.** prior to (5 ng/ml) IL-6 (a-b) or PBS (c) treatment. The expressions of MMP-3 and MMP-13 were determined by western blot (a) and RT-qPCR (b-c). The relative band intensity of MMP-3/MMP-13 in western blot was quantified using IMAGE J software and indicated under each lane. The data are presented as the mean ± SEM, *n* = 3. **p < 0.01, ***p < 0.001, One-way ANOVA with post Newman-Keuls test. One representative from three experiments is shown. CGK733 and BAY11–7082 were shown as CGK and BAY, respectively, for limited space in the figure.

To further explore the role of ATM phosphorylation in IL-6 augmenting lung cancer metastasis, a panel of lung cancer cells was conferred the inhibition of ATM/p65 and the MMPs' expressions were monitored. While the usage of ATM/p65 inhibitors abrogated IL-6 increasing MMP-3/MMP-13 expression in protein level (Figure 5a), the silencing of ATM/p65 abolished IL-6's effect on MMP-3/MMP-13 expression in mRNA level (Figure 5b). Importantly, the suppression of ATM/p65 in LTEP-a-2 and NCI-H520 cells also decreased high IL-6 level correlating MMP-3/MMP-13 expression (Figure 5c). Apart from MMP-3 and MMP-13, MMP-1/MMP-2 expression was obviously decreased by the inhibition of ATM/p65 (Supplementary Figure S5c–S5d). All together, these data suggest that ATM could be activated by the treatment with IL-6, hence increases the expression of MMP-3/MMP-13 and promotes lung cancer metastasis.

### Inhibition of ATM activation abrogates IL-6 increasing lung cancer metastasis *in vivo*

**Figure 6:**
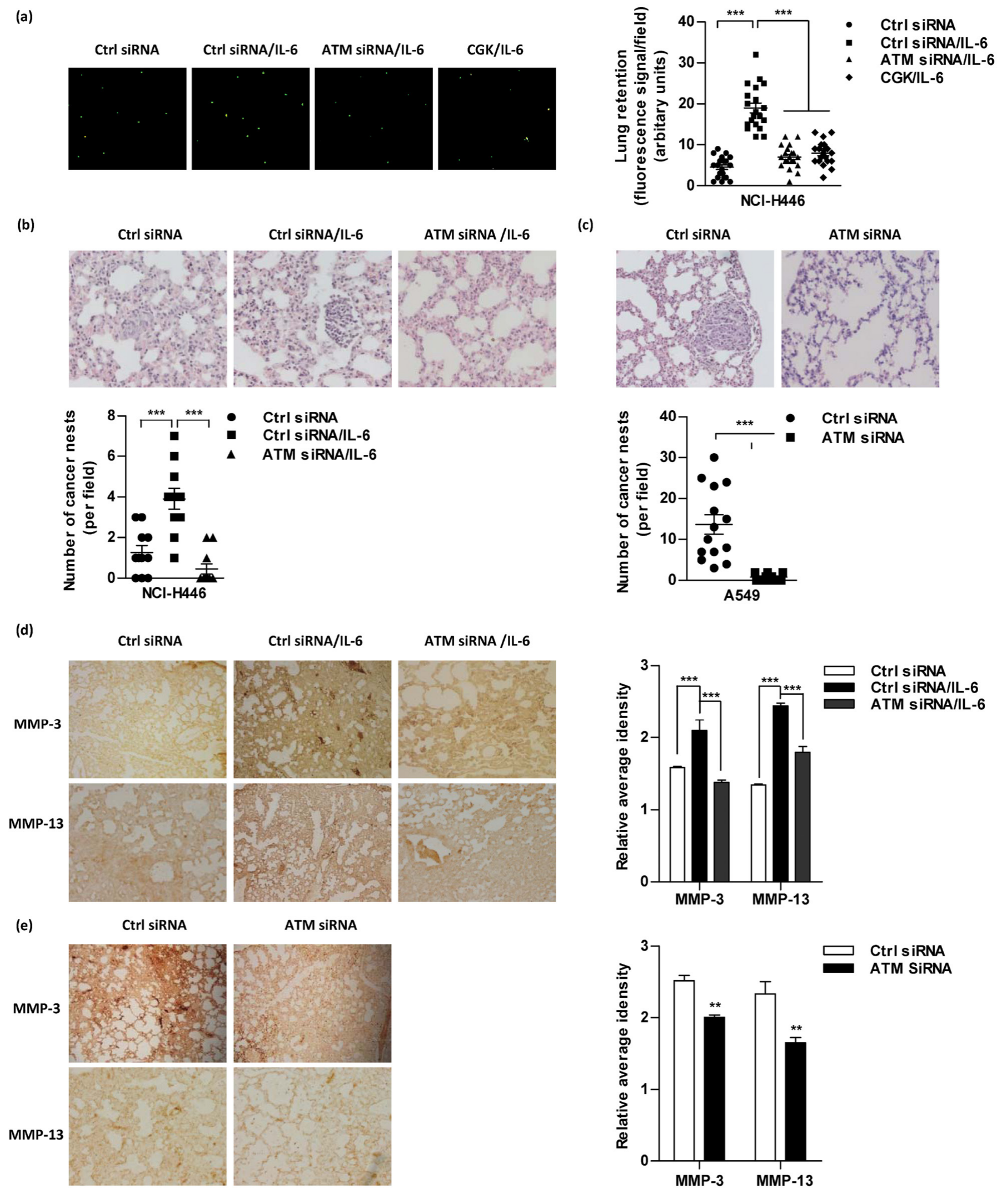
Inhibition of ATM phosphorylation abrogates IL-6 increasing lung cancer metastasis *in vivo* **a.** For lung retention assays, 5 × 10^5^ NCI-H446 cells pretreated with IL-6, siRNA or related kinase inhibitors were labeled with CFSE and then injected to Balb/c nude mice (5–6 weeks old) through the tail vein (*n* = 4 per group). Lung retention is represented as fluorescence signals (CFSE-Green from lung epithelium cells in left panel). Dots represent NCI-H446 cells retained in the lungs 10 h post-tail vein injection (right panel). 10 fields per mouse were analyzed. **b–e.** For lung cancer metastasis model, 8 × 10^5^ NCI-H446 (b, d) or A549 (c, e) cells were conferred ATM siRNA transfection and transferred to Balb/c nude mice (5–6 weeks old) through tail vein (*n* = 4 per group). NCI-H446 cells transferred mice were further subcutaneously conferred IL-6 administration. 2 weeks after adoptive transfer, the lungs were dissociated and performed H&E (b-c) and Immunohistochemistry staining (d-e). The number of cancer nests in lungs was counted; 11–13 slides per condition (b-c). The signals of MMP-3 and MMP-13 were quantified using the Image-Pro Plus software (d-e); 3 fields per condition.

Tail vein injection is a widely accepted method to measure metastatic potential and the ability of cancer cells to attach to blood vessels and survive in the lung [21]. Similar amounts of lung cancer cells suffered from ATM inhibition anchored in the vessels of the lungs (data not shown). Despite IL-6 treatment increased cell retention in the lungs, the inhibition of ATM obviously abrogated IL-6's effect on lung cancer cell retention (Figure 6a), indicating that ATM phosphorylation facilitates lung cancer cell metastasis. To address this issue, *in vivo* lung cancer metastasis test was performed. Hematoxylin and eosin sections revealed nest cells with prominent and irregular nuclei (Figure 6b–6c, Figure S6a–S6b). The number of cancer nests in the lung represents the proximal colonization of the cancer cells (Figure 6b–6c), while the metastatic nodes in the liver represent the remote metastasis (Figure S6a–S6b). Importantly, the inhibition of ATM abrogated IL-6's effect on cancer nests formation in the lung of NCI-H446 or A549 cells transferred recipients (Figure 6b–6c). Moreover, the expressions of MMP-3/MMP-13 in both lung (Figure 6d–6e) and liver (Supplementary Figure S6c–S6d) were also suppressed by the inhibition of ATM. As the silencing of ATM/p65, IL-6, MMP-3/MMP-13 efficiently abrogated ATM phosphorylation (Supplementary Figure S7) and gene expression (Supplementary Figure S8), the above observations demonstrate that high level of ATM phosphorylation contributes to IL-6 correlating MMP-3/MMP-13 expression and lung cancer metastasis *in vivo*.

## DISCUSSION

Our previous studies demonstrate that ATM phosphorylation [25] and elevated IL-6 level [11] contribute to chemotherapeutic agents-inducing MDR formation in lung cancer. The present study demonstrated that high IL-6 level increases both expressions and activities of MMP-3 and MMP-13, hence augments cell migration abilities of lung cancer (Figures 1–3). Moreover, the inhibition of ATM phosphorylation not only abrogates IL-6 increased, MMP-3/MMP-13 mediated cell migration *in vitro* (Figure 4–5), but also suppresses IL-6 correlating lung cancer metastasis *in vivo* (Figure 6).

The degradation of ECM and basement membrane by MMPs is a critical process in tumor invasion and metastasis [27]. Although down-regulation of MMP-3 reduced lung cancer spontaneous metastasis [28], genetic ablation of MMP-3 did not significantly affect breast cancer metastasis [29] was also documented. MMP-13 abrogation was demonstrated to reduce breast cancer metastases by inhibiting osteoclast cells' differentiation [30] or decreasing stromal MMP-13 expression [31]. While MMP-13 replenishment increased tumor metastasis by promoting angiogenesis [32], MMP-13 inactivation inhibited stromal-promoting melanoma metastasis [33], indicating that the interactions of tumor and stromal cells might be an important issue for MMP-13 participating in metastasis. Here, we investigated the role of ATM phosphorylation in IL-6 increasing MMP-3/MMP-13 expression, but the exact mechanism by which MMP-3/MMP-13 promoting lung cancer metastasis is still to be clarified.

As the MMP family is comprised of more than 25 related zinc-dependent enzymes [34], the findings that MMP-3/MMP-13 is involved in ATM activation increasing cell migration (Figures 2–6) and IL-6 increases MMP-1/MMP-2 expression (Supplementary Figure S2) cannot exclude the possibility that other MMPs might also contribute to inflammation-associated lung cancer metastasis. Meanwhile, we also mention that EMT contributes to tumor progression and metastasis [35–37]. Apart from transforming growth factor beta (TGF-β) [38], IL-6 has been documented to be involved in lung cancer EMT via signal transducer and activator of transcription 3 (STAT3) [39], Notch [40–41] pathway. As EMT could be affected by the composition and structure of ECM [42], the findings that ATM phosphorylation increases MMP-3/MMP-13 expression and promotes lung cancer metastasis indicate that the phosphorylation of ATM might be involved in IL-6 promoting EMT. The exact effects and mechanism of ATM activation on IL-6 increasing EMT require further investigation.

STAT3 has been documented to be involved in tumor growth and metastasis in many types of tumor [43–50]. Once binding to IL-6 receptor (IL-6R) and gp130 receptor, IL-6 could activate STAT3, mitogen-activated protein kinase (MAPK) and phosphatidylinositol 3-kinase (PI3K) pathways and increase tumor metastasis [43–44, 51], indicating that IL-6 increasing metastasis is IL-6R/gp130 dependent. Meanwhile, a novel mechanism that IL-6 promoted prostate cancer metastases through a soluble IL-6 receptor (sIL-6R) without activation of STAT1, STAT3 or MAPK was also documented [52]. In the present study, despite ATM phosphorylation was achieved by IL-6 treatment, the exact mechanism that IL-6 inducing ATM activation still needs further investigation.

The migration and invasion characteristics that are related to inflammatory response play important roles in the development of lung cancer. While Erk1/2-NF-κB pathway was reported to be partially involved in inflammatory factors TGF-β1, TNF-α and IL-6 correlated lung cancer invasion [53], p38-NF-κB and STAT3-NF-κB pathways were demonstrated to inhibit miR-365 expression and regulate IL-6 repressing miR-98 levels respectively [54–55]. All these studies indicate that Erk1/2, p38 and STAT3 are up-stream molecules of NF-κB. The activation of ATM-NF-κB increasing MDR associated genes expression [11] indicates that ATM is another up-stream kinase of NF-κB. Hence, it was not surprise to find that the inhibition of NF-κB pathway was more efficient than that of ATM in abolishing IL-6's effects on MMP-3/MMP-13 expression and cell migration (Figure 4, 5). As the STAT3 phosphorylation at Ser(727) is triggered by active RSK2 or JNK1 in the presence of intracellular phosphorylation process of ATM [56], the exact crosstalk between ATM and other NF-κB upstream molecules in IL-6 correlated lung cancer metastasis still need further exploration.

ATM, which expresses in a variety of tumor, is commonly considered as a tumor suppressor for its role in DNA damage repair machinery [57–63] and in SATB1-induced tumorigenic progression [64]. In the present study, our results reveal that IL-6 inducing ATM phosphorylation increases lung cancer metastasis via up-regulation of MMP-3/MMP-13. Consistent to our study, high phosphorylation level of ATM decrease disease-free survival was also documented [65]. Hence, there is still controversial about the role of ATM, to suppress or activate tumor progression. As an early DNA damage-response kinase, ATM might immediately repair SATB1-induced chromatin remodeling, inhibit genetic alterations, and suppress malignant transformation. Therefore, it is no surprise to find that ATM abundant breast epithelial cells, but not ATM depletion cells, were resistant to SATB1-induced malignant progression [64]. On the other hand, the phosphorylation of ATM can also be induced by IL-6 treatment without obvious DNA double break [11]. Similar to the present study, hypoxia treatment increasing ATM phosphorylation without apparent DNA damage was demonstrated to promote breast cancer metastasis via NF-κB pathway [66]. Thus, the high phosphorylation level of ATM contributing to IL-6 correlated lung cancer metastasis indicates that the functions of ATM might be dependent on cell stress, DNA damage and signaling networks.

Taken together, our results reveal for the first time that the inhibition of ATM phosphorylation efficiently decreases the expressions of MMP-3/MMP-13 and inhibits IL-6 correlated lung cancer metastasis, indicating that the inhibition of ATM phosphorylation is a potential strategy for dealing with inflammation correlating lung cancer metastasis.

## MATERIALS AND METHODS

### Reagents

Recombinant human or mouse IL-6 and Human IL-6 Module Set ELISA kit were obtained from eBioscience (San Diego, CA, USA). Antibodies for western blot and BAY11–7082 were purchased from Cell Signaling Technology (Beverly, MA, USA) and Abcam (San Francisco, CA, USA). ATM inhibitor CGK733, MMPs inhibitor Z-Pro-Leu-Gly-hydroxamate (HYD), 3B-3CT and NNGH was acquired from Sigma (Shanghai, China). MMP-3 inhibitor UK-356618 was from Toronto Research Chemicals (North York, Canada). The siRNA of IL-6, ATM, p65 and controls was purchased from Santa Cruz Biotechnology (Dallas, TX, USA). SYBR Premix Ex Taq, Trizol and Prime-Script Reverse Transcriptase were obtained from TaKaRa Biotechnology (Dalian, China). Transwell compartment was from Corning (New York city, NY, USA). RPMI-1640, DMEM and fetal bovine serums were acquired from Hyclone (Logan, UT, USA). UltraSensitiveTM SP IHC Kit was from Maixin Biotech (Fuzhou, China). Human MMP-3 and MMP-13 ELISA kits were from Shanghai Huiying Biological Technology (Shanghai, China). Lipofectamine 2000 and carboxyfluorescein diacetate succinimidyl ester (CFSE) were bought from Invitrogen (Eugene, OR, USA).

### Cell culture, cell lines and siRNA transfection

Human small cell lung cancer (SCLC) NCI-H446 cells, non-small cell lung cancer (NSCLC) NCI-H1299 cells, lung adenocarcinoma LTEP-a-2 cells and squamous cell carcinoma NCI-H520 cells were obtained from Type Culture Collection of the Chinese Academy of Sciences (Shanghai, China). Human lung carcinoma A549 cells were kindly provided by Professor GH. Jin (Xiamen University). All the cells were grown in RPMI-1640 or DMEM medium containing 10% FBS. For siRNA transfection, 30–50% confluent cells were transfected with siRNA using Lipofectamine 2000. The cells were harvested 48 h after transfection. The final concentration for siRNA is 100 nM. The silence effects of indicative siRNA in NCI-H446 cells were validated in Supplementary Figure S8.

### Animals

Pathogen-free BALB/c nude mice (5–6 weeks old, female) were bought from the Shanghai Laboratory Animal Center of Chinese Academy of Sciences and kept at the Animal Center of Xiamen University. The protocol was approved by the Committee on the Ethics of Animal Experiments of the Xiamen University.

### RT-qPCR

Endogenous IL-6 expression of lung cancer cell lines and MMPs expression were investigated by RT-qPCR analysis. Briefly, the cellular RNA was extracted, and reverse transcription was performed using PrimeScript Reverse Transcriptase. Quantitative PCR analysis was performed using ABI 7000 Sequence Detection System in the presence of SYBR Green. The cycling parameters were 95°C for 3 min, followed by 40 cycles of 95°C for 5 s, 60°C for 30 s, and 72°C for 60 s, with a final extension at 72°C for 10 min; a melting curve analysis was subsequently conducted. Each assay was performed in triplicate, and the relative expression levels (defined as fold changes) of the target genes were normalized to the folds of corresponding control cells. The PCR primer sequences are listed in Table 1.

**Table 1:** Real-time PCR primer sequence

Genes	F/R	Sequence
β-actin	F	5′-TCAAGATCATTGCTCCTCCTG-3′
β-actin	R	5′-CTGCTTGCTGATCCACATCTG-3′
IL-6 (F)	F	5′-CCACACAGACAGCCACTCACC-3′
IL-6 (F)	R	5′-CTACATTTGCCGAAGAGCCCT-3′
MMP-3	F	5′-CCTGCTTTGTCCTTTGATGC-3′
MMP-3	R	5′-TGAGTCAATCCCTGGAAAGTC-3′
MMP-13	F	5′-TTGTTGCTGCGCATGAGTTCG-3′
MMP-13	R	5′-GGGTGCTCATATGCAGCATCA-3′

### ELISA

To detect the release of IL-6 in corresponding lung cancer cell lines and determine MMP-3/MMP-13 activity, 1 × 10^5^ cells plated in 24-well plates were treated with PBS or IL-6 (5 ng/ml) for 24 h and the supernatant was collected. The IL-6 concentration or the MMP-3/MMP-13 activity was determined by enzyme double-antibody indirect immunoassays with respective ELISA kits in accordance with manufacturer protocol [67].

### Western blot

NCI-H446 cells were treated with IL-6 (5 ng/ml) for indicated periods or pretreated with inhibitors prior to IL-6 48 h stimulation. The expressions of MMP-3 and MMP-13 were determined via western blot analysis. The expression of Δ-actin was used as a loading control. The relative band intensity of western blot was quantified using IMAGE J software and indicated under each lane.

### Transwell migration assay

Cell migration ability was determined via transwell migration assay [22]. Briefly, 4 × 10^3^ cells pretreated with IL-6, siRNA or related kinase inhibitors were seeded onto the top chamber of Transwell inserts (24-well insert, 8 μm pore size; Corning) in serum-free-medium. Culture medium with 10% fetal bovine serum in the lower compartment was used as a chemo attractant. After 18 h incubation, the cells on the upper surface of the membrane were wiped out using a cotton swab. Then the cells that migrated to the lower surface were fixed and stained. Cell migration ability was determined by calculating the number of migrated cells in three visual fields per well by microscopy with 100 × magnification.

### *In vivo* lung retention assays

Early migration ability was determined via lung retention assays [21]. Briefly, 5 × 10^5^ NCI-H446 cells pretreated with IL-6, siRNA or related kinase inhibitors were labeled with CFSE (1:1000 dilution in PBS), trypsinized and injected into BALB/c nude mice through the tail vein in 200 μl saline. Mice were sacrificed after 1 h (to show the early arrive at the lung) and 10 h. The lungs were washed, fixed and examined for fluorescently labeled cells under fluorescence microscope. Lung retention is represented as fluorescence dots per field, and 10 fields per mouse were analyzed. Four mice per condition were conducted.

### *In vivo* lung cancer metastasis test

Late migration ability was determined via murine lung cancer metastasis test [21]. Briefly, NCI-H446 or A549 cells were transfected with ATM siRNA. 48 h post-transfection, 8 × 10^5^ cells per mouse were transferred to BALB/c nude mice in 100 μl saline through the tail vein. The mice were further subcutaneously conferred IL-6 administration, once a day. The dose of IL-6 was 1.5 μg/kg for first 3 days, then 2.5 μg/kg throughout next 3 days and reached 5 μg/kg over next 7 days. 2 days after the last injection, the lungs and livers were dissociated and preserved for further studies. Each experiment had four mice per condition.

### H&E and immunohistochemistry staining

*In vivo* cell migration ability and the expression of MMP-3/MMP-13 in lung were determined via H&E and Immunohistochemistry staining [68]. Briefly, free-floating lung sections (4 μM) were obtained using a slicing system (Leica RM2135). For H&E staining, the sections were stained with hematoxylin, then counterstaining with eosin. For immunohistochemistry staining, endogenous peroxidase activity was quenched in H_2_O_2_, and 90% formic acid min was used to expose epitope. The primary antibody was applied overnight at 4°C and then biotinylated secondary antibody at room temperature. After a final wash, the slides were developed with diaminobenzidine substrate by using the avidin-biotin HRP system. The signals were quantified using the Image-Pro Plus software.

### Statistical analysis

All experiments were repeated at least three times to confirm the similar results. Data were presented as the mean ± SEM. Student's *t* test or one-way ANOVA with the post Newman-Keuls test was applied. Statistical differences were considered to be significant at *p* < 0.05.

## SUPPLEMENTARY FIGURES


