# Supplementary material for: Interleukin 6 trigged ataxia-telangiectasia mutated activation facilitates lung cancer metastasis via MMP-3/MMP-13 up-regulation
Source: Oncotarget. 2015 Oct 22;6(38):40719–33. doi: 10.18632/oncotarget.5825 (PMC4747364; doi:10.18632/oncotarget.5825)
Supplement: Supplementary file 1 [file oncotarget-06-40719-s001.pdf]

SUPPLEMENTARY FIGURES

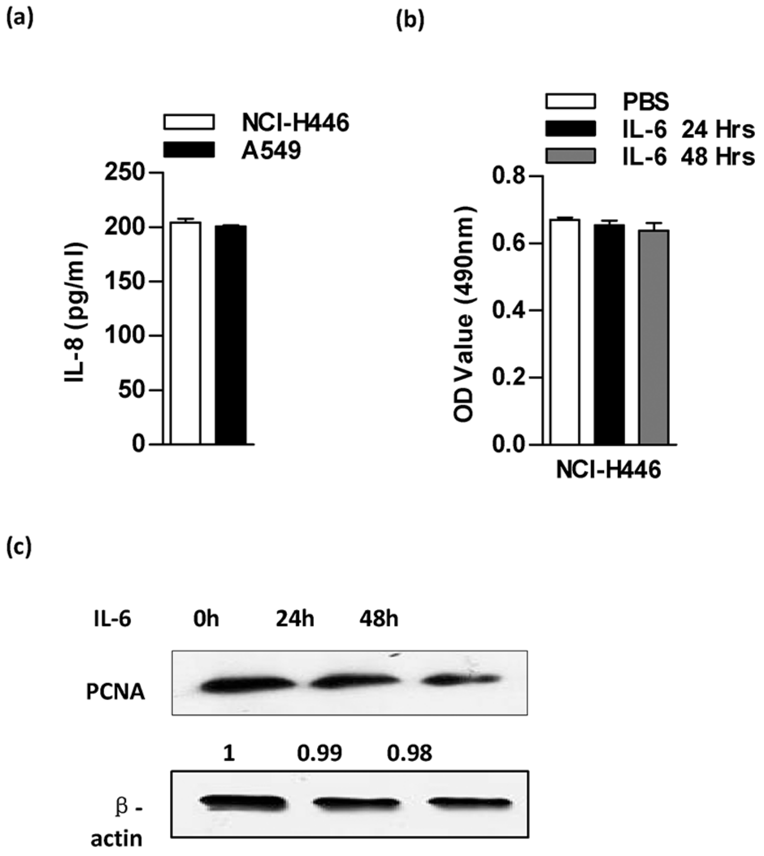

Supplementary Figure S1: IL-6 increasing lung cancer cell migration is non-relevant to cell proliferation. The level of IL-8 in supernatant of NCI-H446 and A549 cells was determined by ELISA **a.** NCI-H446 cells were treated with IL-6 (5 ng/ml) and cell proliferation was determined by MTT assay **b.** and PCNA western blot **c.** The relative band intensity of PCNA western blot was quantified using IMAGE J software and indicated under each lane.

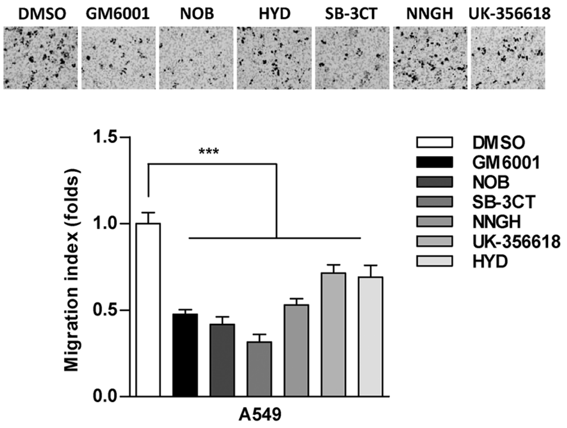

Supplementary Figure S2: MMPs are involved in cell migration in A549 cells. A549 cells were conferred MMPs inhibitors treatment and cell migration was determined by Transwell migration assay. The data are presented as the mean ± SEM, $n = 3$. *** $p < 0.001$, One-way ANOVA with post Newman-Keuls test. One representative from three experiments is shown.

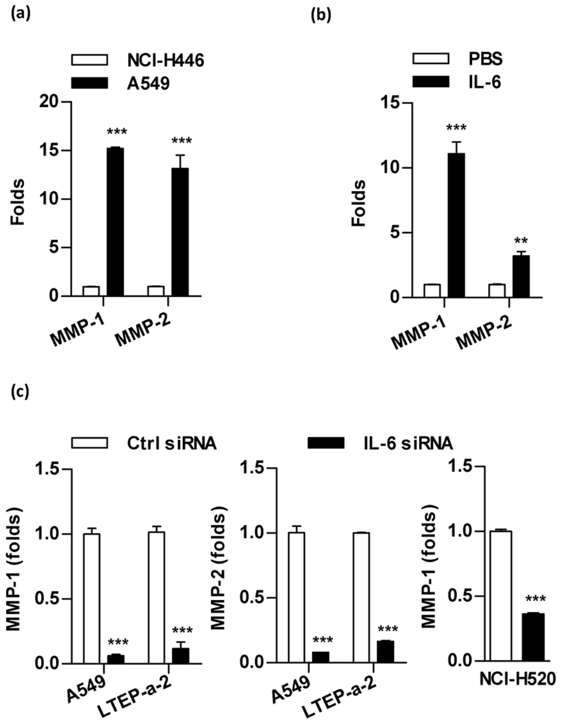

Supplementary Figure S3: IL-6 increases MMP-1/MMP-2 expression in lung cancer cells. NCI-H446, A549, LTEP-a-2 and NCI-H520 cells were treated with PBS **a.** IL-6 (5 ng/ml) **b.** or IL-6 siRNA transfection **c.** The expression of MMP-1/MMP-2 was determined by RT-qPCR. The data are presented as the mean ± SEM, $n = 3$. $^{**}p < 0.01$, $^{***}p < 0.001$, Student *t* test. One representative from three experiments is shown.

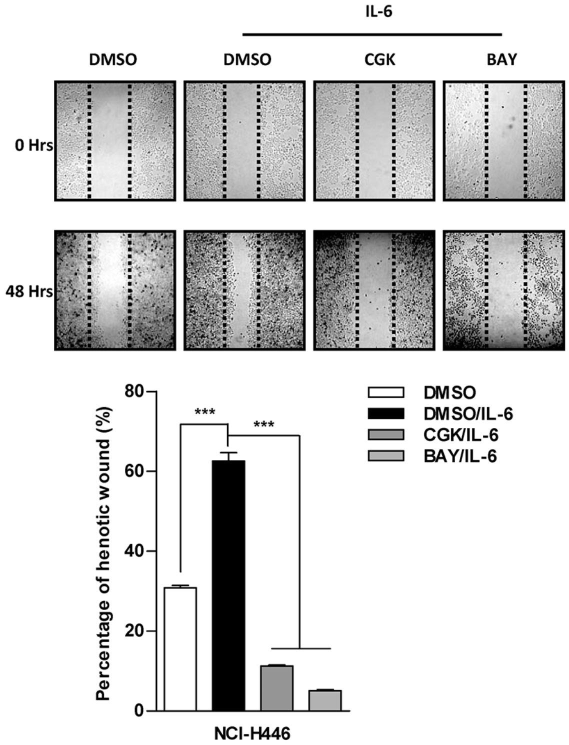

Supplementary Figure S4: Inhibition of ATM activity abolishes IL-6's effect on cell migration in NCI-H446 cells. NCI-H446 cells were pretreated with CGK733 or BAY11–7082 prior to IL-6 (5 ng/ml) stimulation. Cell migration was determined by wound healing assay. $^{***}p < 0.001$, One-way ANOVA with post Newman-Keuls test. One representative from three experiments is shown. CGK733 and BAY11–7082 were shown as CGK and BAY, respectively, for limited space in the figure.

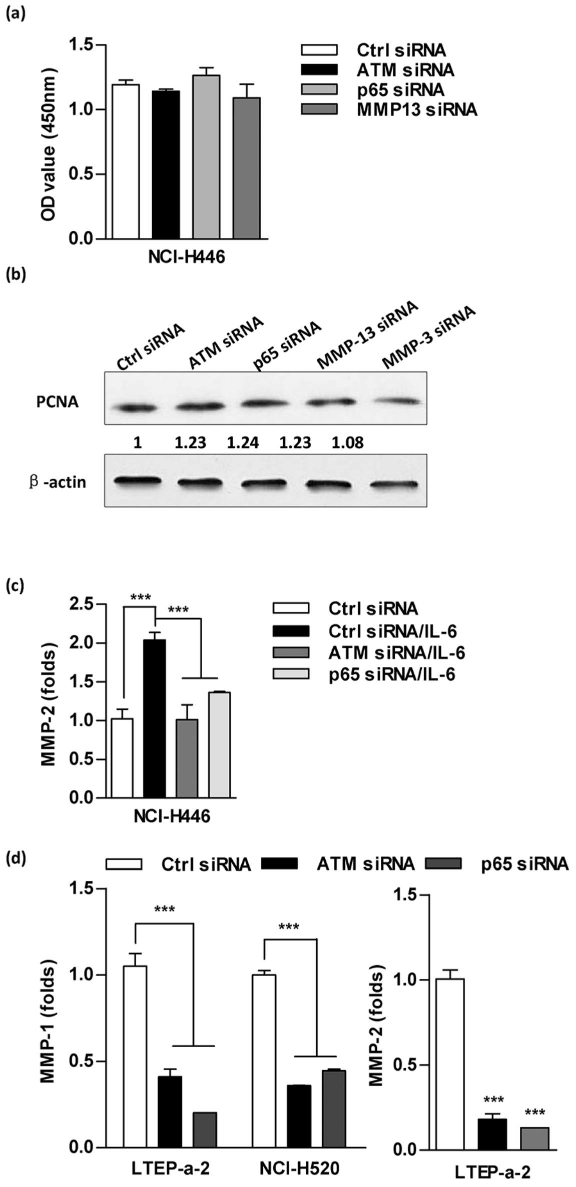

Supplementary Figure S5: Inhibition of ATM and NF-κB activation abrogates the effect of IL-6 on MMP-1/MMP-2 up-regulation. a–b. NCI-H446 cells were treated with indicative siRNA transfection and cell proliferation was determined by MTT assay (a) and PCNA western blot (b). The relative band intensity of PCNA western blot was quantified using IMAGE J software and indicated under each lane. **c–d.** NCI-H446, LTEP-a-2 or NCI-H520 cells were pretreated with siRNA transfection prior to IL-6 (5 ng/ml) (c) or PBS (d) treatment. The expressions of MMP-1/MMP-2 were determined by RT-qPCR. The data are presented as the mean ± SEM, $n = 3$. $^{**}p < 0.01$, $^{***}p < 0.001$, One-way ANOVA with post Newman-Keuls test. One representative from three experiments is shown.

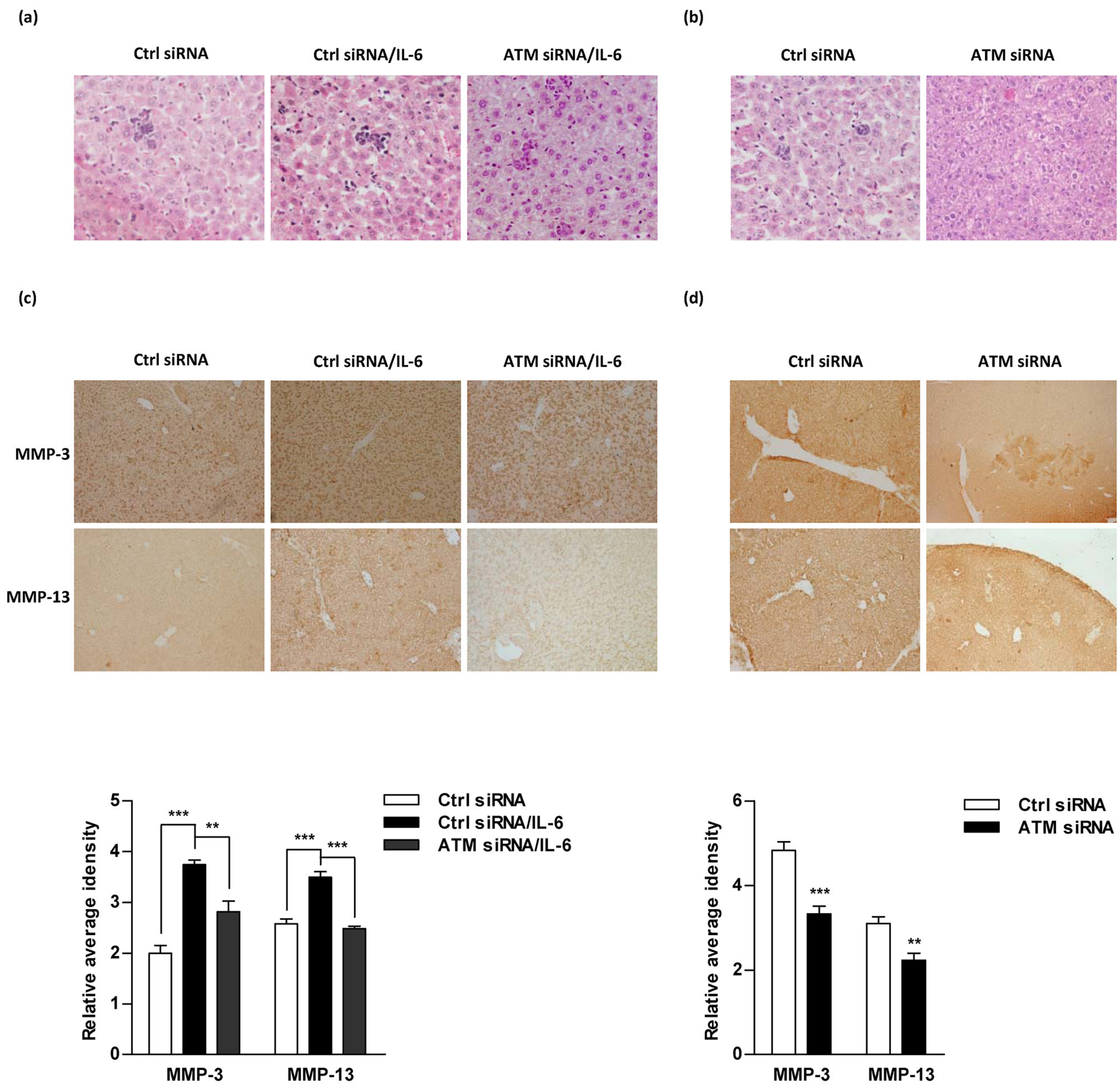

Supplementary Figure S6: Inhibition of ATM activation abrogates IL-6 increasing MMP-3/MMP-13 expression in liver of the recipient. 8×10^5 NCI-H446 **a, c.** or A549 **b, d.** cells were conferred ATM siRNA transfection and transferred to Balb/c nude mice (5–6 weeks old) through tail vein (n = 4 per group). NCI-H446 cells transferred mice were further subcutaneously conferred IL-6 administration. 2 weeks after adoptive transfer, the livers were dissociated and performed H&E (a-b) and Immunohistochemistry staining (c-d). The signals of MMP-3 and MMP-13 were quantified using the Image-Pro Plus software; 3 fields per condition.

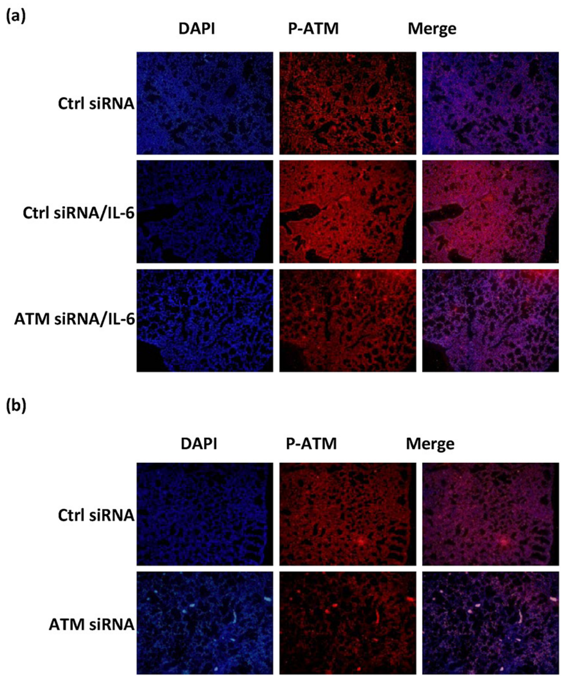

Supplementary Figure S7: Inhibition of ATM abrogates IL-6 effect on ATM phosphorylation *in vivo*. 8 × 10^5 NCI-H446 **a.** or A549 **b.** cells were conferred ATM siRNA transfection and transferred to Balb/c nude mice through tail vein. NCI-H446 cells transferred mice were further subcutaneously conferred IL-6 administration. 2 weeks after adoptive transfer, the lung were dissociated and performed Immunofluorescence staining.

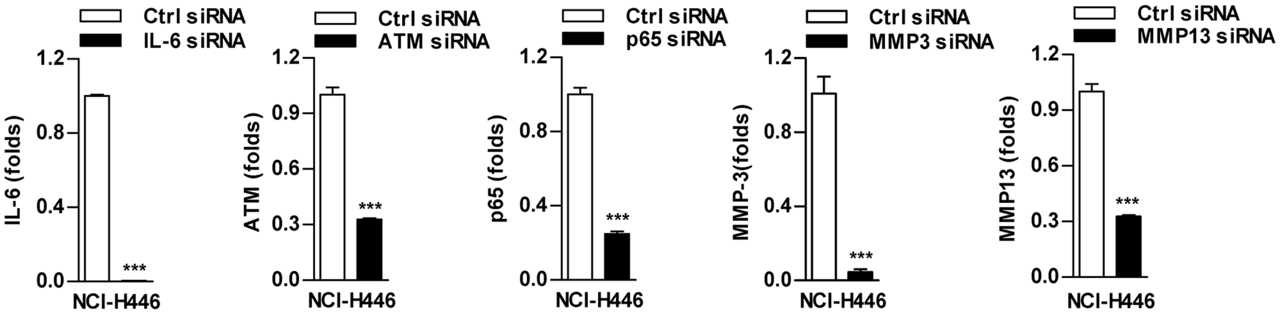

Supplementary Figure S8: The silence effects of siRNA in NCI-H446 cells. NCI-H446 cells were treated with indicative siRNA transfection and the silence effects of relative gene transcription were validated by RT-qPCR. The data are presented as the mean ± SEM, $n = 3$. $^{**}p < 0.01$, $^{***}p < 0.001$, One-way ANOVA with post Newman-Keuls test. One representative from three experiments is shown.
